
JOURNAL INFORMATION
==============================
NLM Title Abbreviation: Access Microbiol
Journal ID: Access Microbiol
NLM Journal ID: 101746927
Journal ID: acmi

Access Microbiology

EISSN: 2516-8290
Publisher: Microbiology Society

ARTICLE INFORMATION
==============================
PMCID: PMC7544069
PMID: 33062931
Article version: 1
Subjects: Abstracts from the Microbes in Medicine Meeting 2019

Poster Presentations

Publication date: 2020
Electronic publication date: 2020 Jan 31
Volume: 2
Issue: 1
Electronic Location ID: po0007-po0028
Copyright: © 2020 The Authors
Copyright year: 2020
License: This is an open-access article distributed under the terms of the Creative Commons Attribution License.
License URL: https://creativecommons.org/licenses/by/4.0/

==============================

DOI: 10.1099/acmi.mim2019.po0007

Defining the link between efflux pumps and biofilm formation

http://jmmcr.microbiologyresearch.org

Holden Emma *
Webber Mark
Quadram Institute , Norwich , United Kingdom
*Correspondence: Emma Holden, emma.holden@quadram.ac.uk

DOI: 10.1099/acmi.mim2019.po0008

Carbapenem-resistant Enterobacteriaceae : A serious concern in cancer patients

http://jmmcr.microbiologyresearch.org

Biswas Sanjay *
Bhat Vivek
Kelkar Rohini
Tata Memorial Center , Mumbai , India
*Correspondence: SANJAY BISWAS, skbiswas67@rediffmail.com

DOI: 10.1099/acmi.mim2019.po0009

The (p)ppGpp-binding GTPase Era promotes rRNA processing and cold adaptation in Staphylococcus aureus

http://jmmcr.microbiologyresearch.org

Wood Alison
Irving Sophie
Bennison Daniel
Corrigan Rebecca *
University of Sheffield , Sheffield , United Kingdom
*Correspondence: Rebecca Corrigan, r.corrigan@sheffield.ac.uk

DOI: 10.1099/acmi.mim2019.po0011

The antibiofilm effects of docosahexaenoic acid

http://jmmcr.microbiologyresearch.org

Brady Jonathan *
TUD , Tallaght campus , Dublin
*Correspondence: Jonathan Brady, jonathan.brady@postgrad.it-tallaght.ie

DOI: 10.1099/acmi.mim2019.po0012

Antimicrobial resistance in bacteria associated with childhood pneumonia in Malawi

http://jmmcr.microbiologyresearch.org

Moody Mitchell *
TUD Tallaght Campus , Dublin , Ireland
*Correspondence: Mitchell Moody, mitchell.moody@postgrad.it-tallaght.ie

DOI: 10.1099/acmi.mim2019.po0013

Significance of the chromosomal positions of the genes encoding DNA gyrase in Salmonella enterica Serovar Typhimurium

http://jmmcr.microbiologyresearch.org

Pozdeev German *
Trinity College Dublin , Dublin , Ireland
*Correspondence: German Pozdeev, pozdeevg@tcd.ie

DOI: 10.1099/acmi.mim2019.po0014

Understanding selection of antimicrobial resistance in biofilms through experimental evolution

http://jmmcr.microbiologyresearch.org

Wickham Gregory *
Trampari Eleftheria
Webber Mark
Quadram Institute , Norwich , United Kingdom
Norwich Medical School , University of East Anglia , Norwich
*Correspondence: Gregory Wickham, gregory.wickham@quadram.ac.uk

DOI: 10.1099/acmi.mim2019.po0016

The evolution of Pseudomonas aeruginosa during short-term acute respiratory infections

http://jmmcr.microbiologyresearch.org

Wheatley Rachel *
Kapel Natalia
Caballero Julio Diaz
Paling Fleur
Sifakis Frangiscos
Ruzin Alexey
Timbermont Leen
Kluytmans Jan
Malhotra Surbhi
MacLean Craig
University of Oxford , Oxford , United Kingdom
University Medical Centre Utrecht , Utrecht , Netherlands
AstraZeneca , Gaithersburg , USA
University of Antwerp , Antwerp , Belgium
*Correspondence: Rachel Wheatley, rachel.wheatley@zoo.ox.ac.uk

DOI: 10.1099/acmi.mim2019.po0017

Plasma activated liquids: New decontamination solutions

http://jmmcr.microbiologyresearch.org

Tsoukou Evanthia *
Bourke Paula
Boehm Daniela
Plasma Research Group , School of Food Science and Environmental Health , Technological University Dublin
*Correspondence: EVANTHIA TSOUKOU, evatsoukou@yahoo.com

DOI: 10.1099/acmi.mim2019.po0018

A matter of life and death; identifying host genomic factors that determine susceptibility of chickens to highly pathogenic avian influenza

http://jmmcr.microbiologyresearch.org

Donnelly Cal *
Fulton Janet E.
Wolc Anna
Drobik-Czwarno Wioleta
Digard Paul
Smith Jacqueline
Roslin Institute , Edinburgh , United Kingdom
Hy-Line International , Iowa , USA
Warsaw University of Life Sciences , Warsaw , Poland
*Correspondence: Cal Donnelly, cal.donnelly@roslin.ed.ac.uk

DOI: 10.1099/acmi.mim2019.po0019

Characterising the urinary microbial community in patients with symptoms of urinary tract infection

http://jmmcr.microbiologyresearch.org

Sathiananthamoorthy Sanchutha *
Malone-Lee James
Spratt David
Rohn Jennifer
University College London , London , United Kingdom
*Correspondence: Sanchutha Sathiananthamoorthy, rebmssa@ucl.ac.uk

DOI: 10.1099/acmi.mim2019.po0020

Vorinostat (SAHA) promotes innate and adaptive immunity to Mycobacterium tuberculosis

http://jmmcr.microbiologyresearch.org

Cox Donal *
Coleman Amy
Gogan Karl
Dunne Padraic
Basdeo Sharee
Keane Joseph
Trinity College , Dublin , Ireland
Royal College Of Surgeons , Dublin , Ireland
*Correspondence: Donal Cox, coxdo@tcd.ie

DOI: 10.1099/acmi.mim2019.po0021

Development of a microbially-derived therapy against Fusobacterium nucleatum, a bacterial pathogen linked with colorectal cancer

http://jmmcr.microbiologyresearch.org

Lawrence Garreth *
McCarthy Niamh
Begley Máire
Cotter Paul
Guinane Caitriona
Department of Biological Sciences , Cork Institute of Technology , Cork
Teagasc , Food Research Centre , Moorepark
APC Microbiome Institute , University College Cork , Cork
*Correspondence: Garreth Lawrence, garreth.lawrence@mycit.ie

DOI: 10.1099/acmi.mim2019.po0022

Quorum quenching activity of Aerva lanata against Catheter Associated Urinary Tract Infections (CAUTI) Pathogens

http://jmmcr.microbiologyresearch.org

Mohanvel Sucharitha Kannappan *
Rajasekharan Satish Kumar
Department of Biotechnology , D.G. Vaishnav College , Chennai
Center for Advanced Studies in Botany , University of Madras , Chennai
Centre for Research and Development , PRIST University , Chennai
*Correspondence: Sucharitha Kannappan Mohanvel, charusunder@gmail.com

DOI: 10.1099/acmi.mim2019.po0023

Salmonella antibiotic persistence and macrophage polarisation

http://jmmcr.microbiologyresearch.org

Majstorovic Andrea *
Hill Peter W. S.
Helaine Sophie
Imperial College London , London , United Kingdom
*Correspondence: Andrea Majstorovic, a.majstorovic18@imperial.ac.uk

DOI: 10.1099/acmi.mim2019.po0024

Whole genome sequencing reveals genetic diversity in Mycobacterium avium subspecies paratuberculosis population circulating in Irish cattle

http://jmmcr.microbiologyresearch.org

Perets Viktor *
Cassidy Sophie
Kenny Kevin
O'Connor Aoife
O'Mahony Jim
Crispell Joseph
Gordon Stephen
UCD School of Veterinary Medicine , University College Dublin , Dublin
Central Veterinary Research Laboratory , Backweston , Co. Kildare
Department of Biological Sciences , Cork Institute of Technology , T12 P928 Cork
*Correspondence: Viktor Perets, viktor.perets@ucdconnect.ie

DOI: 10.1099/acmi.mim2019.po0025

Lactate improves killing of Mycobacterium tuberculosis in human macrophages

http://jmmcr.microbiologyresearch.org

Maoldomhnaigh Cilian Ó
Cox Donal *
McQuaid Kate
Gogan Karl
Basdeo Sharee
Keane Joseph
Trinity College , Dublin , Ireland
*Correspondence: Donal Cox, coxdo@tcd.ie

DOI: 10.1099/acmi.mim2019.po0026

Using metagenomics to investigate the impact of hospital stay and the ARK intervention on the human gut resistome

http://jmmcr.microbiologyresearch.org

Yokoyama Maho *
Peto Leon
Walker A. Sarah
Llewelyn Martin
Brighton and Sussex Medical School , Brighton , United Kingdom
Nuffield Department of Medicine , Oxford , United Kingdom
*Correspondence: Maho Yokoyama, maho-yokoyama@hotmail.com

DOI: 10.1099/acmi.mim2019.po0027

The molecular basis of antibiotic treatment failure in chronic urinary tract infections

http://jmmcr.microbiologyresearch.org

Fatti Claudio Del *
Jafari Nazila
Hubbard Alasdair
Khasriya Rajvinder
Roberts Adam
Rohn Jennifer
University College London , London , United Kingdom
Liverpool School of Tropical Medicine , Liverpool , United Kingdom
Whittington Hospital Trust , London , United Kingdom
*Correspondence: Claudio Del Fatti, claudio.del-fatti.18@ucl.ac.uk

DOI: 10.1099/acmi.mim2019.po0028

Socially messaging polycellular interactomes for innovative interventions in health and medicine

http://jmmcr.microbiologyresearch.org

Reen F. Jerry *
University College Cork , Cork , Ireland
*Correspondence: Jerry Reen, j.reen@ucc.ie

